# Use of random forest based on the effects of urban governance elements to forecast CO_2_ emissions in Chinese cities

**DOI:** 10.1016/j.heliyon.2023.e16693

**Published:** 2023-06-01

**Authors:** He Zhang, Jingyi Peng, Rui Wang, Mengxiao Zhang, Chang Gao, Yang Yu

**Affiliations:** a Tianjin University, Tianjin, China; b Tsinghua Tongheng Urban Planning & Design Institute, Beijing, China; c Tianjin University Research Institute of Architectural Design & Urban Planning, Tianjin, China

**Keywords:** CO_2_ emissions, Urban governance elements, Random forest, Forecasting

## Abstract

Chinese cities contributes a large amount of CO_2_ emissions. Reducing CO_2_ emissions through urban governance is an important issue. Despite the increasing attention paid on the CO_2_ emission prediction, few studies consider the collective and complex influence of governance element system. To predict and regulate CO_2_ emissions through comprehensive urban governance elements, this paper use the random forest model through the data from 1903 Chinese county-level cities in 2010, 2012 and 2015, and establish a CO_2_ forecasting platform based on the effects of urban governance elements. The results are as follows: (1) The municipal utility facilities element, the economic development & industrial structure element, and the city size &structure and road traffic facilities elements are crucial for residential, industrial and transportation CO_2_ emissions, respectively; (2) Governance elements have nonlinear relationship with CO_2_ emissions and most of the relations present obvious threshold effects; (3) Random forest can be used to predict CO_2_ emissions more accurately than can other predictive models. These findings can be used to conducts the CO_2_ scenario simulation and help government formulate active governance measurements.

## 1 Introduction

Global warming is regarded as one of the greatest concerns confronting humanity today because it causes natural ecological imbalances, harsher weather, and reduced food supply [1]. According to Horton et al. [2], the global average sea level will rise by 1.67–5.61 m by the year 2300, thus posing a serious threat to people's lives and property. Anthropogenic CO_2_ emissions are considered to be the dominant driver of global warming [3]. As the largest developing country, China has produced more than a quarter of the world's total CO_2_ [4]. To cope with the high CO_2_ emissions, urban governance has been emphasized by the Chinese government in recent decades to reduce the absolute demand for energy [5,6]. The Chinese government conducts urban governance mainly through Economic & Social Development Planning and Land & Space Planning. These plannings involved the urban governance contents like social situation, economic development, land use and facilities construction. How to control these urban governance elements to reduce CO_2_ emissions in China has been a very important issue.

Using urban governance elements to predict CO_2_ emissions is important for CO_2_ reduction in China. First, the elements could help decision-makers to deduce future CO_2_ emissions by considering the comprehensive effects of the development of various urban governance elements, thus preliminarily determining whether the dual carbon goals can be achieved based on preconceived planning. Second, the government can formulate effective policies to change the trends of future CO_2_ emissions by controlling the governance elements [7]. It is worth noting that in order to help the government make specific policies, it is necessary to investigate specific CO_2_ emissions sectors and understand how much can be reduced through adjusting the governance elements. Therefore, in the prediction research, the analysis of the influence degree and impact mechanism of governance elements on CO_2_ emission is also essential.

Numerous studies have analyzed the impact of urban governance factors on CO_2_ emissions, laying the foundation for CO_2_ emission prediction and urban governance. However, most of these studies aimed at the correlation between individual governance elements and CO_2_ emissions. In fact, the role of governance factors on CO_2_ emissions is interrelated. The impact of governance factors on CO_2_ emissions is complex [8], and a large number of nonlinear links between governance variables and CO_2_ emissions have not been revealed [9]. Existing studies confirmed the effects of governance elements on CO_2_ emissions. However, the extent of these effects, the specific quantitative relationships between them, and the applications of forecasts have not been adequately addressed in the literature. Furthermore, Although the forecasting of CO_2_ emissions produced by certain departments [10] or regions [11] has been widely developed. However, these studies did not reveal the relationship between governance elements and CO_2_ emissions. Meanwhile, the predictive variables used in the research are socio-economic elements. Other urban governance elements, such as urban space [12] and facilities [13], which have also been confirmed to have significant effects on CO_2_ emissions, were not included in CO_2_ forecasts.

In order to fill the research gap and help the Chinese government to effectively carry out low-carbon urban governance, this paper carries out research on county-level cities in China. The paper aimed to answer 2 main research questions: (1) How to explore the extent of the effects of governance elements on CO_2_ emissions and reveal their non-linear impact relationship? (2) How to predict CO_2_ emission based on the effects of comprehensive urban governance elements? The contributions of this paper can be summarized as follows: (1) We further reveals the relationship between governance elements and CO_2_ emissions, which can help the government to formulate specific planning policies by CO_2_ sectors and governance elements. (2) In addition to socio-economy elements, we comprehensively consider the governance elements including urban space and facilities, and establish a CO_2_ emission prediction model linked to planning policy, which provides a reference for government scenario simulation.

The remainder of this paper is organized as follows. Section 2 reviews the literature about urban governance related to CO_2_ emissions and the models for predicting CO_2_ emissions. Section 3 introduces the data sources, definitions of the governance variables, and basic principles of the random forest. Section 4 discusses the results of the screened-out variables, nonlinear relationships between the governance factors and CO_2_ emissions, and forecasting of CO_2_ emissions. Section 5 summarizes the conclusions and puts forward suggestions for controlling CO_2_ emissions.

## 2 Literature review

### 2.1 Effects of urban governance elements on carbon dioxide emissions

Economic & Social Development Planning and Land & Space Planning conduct urban governance through a series of control elements and variables. Many studies have extensively researched the effects of a variety of urban governance elements on CO_2_ emissions, of which the main sources can be broadly divided into three sectors based on the end-uses of energy: residential, industrial, and transportation [14]. Each CO_2_ emissions sectors have several urban governance elements. The effects of urban governance elements on CO_2_ emissions can be summarized in Table 1.

**Table 1:** Summary of effects of urban governance elements on CO_2_ emissions.

Sector	Urban governance elements	variables and their impact	Research methods	Reference
Residential CO_2_ emissions	City size &structure; Economic development & industrial structure; Municipal utility facilities	Heating facilities	The integrated approach	[15]
		Central heating; natural gas utilization	Panel regression model	[16]
		Population size (+); urbanization (+)	Generalized fisher index	[17]
		Built up areas (+); urbanization (+); population density (+)	Extended STIRPAT model	[18]
		Residential density	An estimation method	[19]
		GDP per capita (“N" shaped curve)	STIRPAT model	[20]
Industrial CO_2_ emissions	City size &structure; Economic development & industrial structure;	Added industrial value	Logarithmic mean divisia index	[21]
		Industrial structure	The spatial econometric technique	[22]
		GDP per capita (+)	LMDI	[23]
		Economic growth; urbanization	Vector autoregressive model	[24]
		Population; built-up areas	Cointegration test	[25]
Transportation CO_2_ emissions	City size &structure; Economic development & industrial structure; Road traffic facilities; Public service facilities	Population	Logarithmic mean divisia index	[26]
		Urbanization	A two-way durbin model	[27]
		Built area; economic development; urban road density	A two-way fixed effect model	[28]
		Public service facilities	GREET Model	[29]
		GDP per capita	Gompertz model	[30]
Total CO_2_ emissions	–	GDP (an inverted-U shape)	Static models and dynamic models	[31]
		GDP (an inverted-U shape)	An econometric approach	[32]
		Added value of primary sector (−)	Autoregressive distributed lag	[33]
		Added value of primary sector	Granger causality estimations	[34]
		Industrial value added per capita (+)	Autoregressive distributed lag	[35]
		Added value of industry (−)	Augmented dickey fuller	[36]

In the residential building sector, the construction of municipal utility facilities relates to heat, gas, electric, and other supply for heating, daily cooking, lighting, and household appliances impact energy consumption in residential buildings. Heating facilities are recognized as a key driver for determining CO_2_ emissions [15]. Cui et al. [16] found that the CO_2_ emissions from centralized heating in the North China Plain had slightly decreased under the “Natural Gas Utilization Policy”, but CO_2_ emissions have continued to grow. In addition, the governance element of city size & structure also deserve attention. Fan et al. [17] proved that population size and urbanization were important pulling factors for the growth of residential CO_2_ emissions in Beijing. Silaydin Aydin et al. [19] believed that residential density affected the formation of residential emissions. Li et al. [18] revealed that residential CO_2_ emissions were positively influenced by built-up areas. Miao et al. [20] tested the environmental Kuznets curve hypothesis by adding the squared and cubed terms of GDP per capita.

For the industrial sector, governance elements of economic development & industrial structure and city size & structure can affect CO_2_ emissions. Ouyang et al. [21] proved a long-term relationship between industrial CO_2_ emissions and added industrial value by using the logarithmic mean divisia index (LMDI) method. Xu et al. [24] showed that economic growth and urbanization had important effects on the CO_2_ emissions of the iron and steel industry. Liu et al. [25] found that adjusting the scales of urban populations and built-up areas were effective ways to achieve low-carbon industrial development. Zhao et al. [22] demonstrated that industrial structure could affect CO_2_ emissions positively. Using the logarithmic mean Divisia index (LMDI) approach, Chen et al. [23] showed that GDP per capita positively influenced industrial CO_2_ emissions growth in China.

For the transportation sector, governance elements like city size & structure, economic development & industrial structure, construction of road traffic facilities and public service facilities are important for the low-carbon governance. Wang et al. [26] used the LMDI to confirm that population size played an important role in increasing CO_2_ emissions. Xu et al. [27] revealed that urbanization level was also one of the driving factors of CO_2_ emissions. Employing a two-way fixed effect model, Yang et al. [28] demonstrated that increases in CO_2_ emissions from transportation could be restrained through the planning of built-up areas, economic development, and urban road density. GDP per capita influenced the CO_2_ emissions from traffic by affecting the number of private cars [30]. Triantafyllidis et al. [29] showed that the locations of public service facilities would affect residents' transportation modes and travel distances, thus affecting CO_2_ emissions from transportation.

In addition to sectoral CO_2_ emissions, the relationship between total CO_2_ emissions and governance elements has also been given considerable attention. Salari et al. [31] found that the relationship between CO_2_ emissions and GDP in the United States was an inverted U-shape. Fujii et al. [32] also observed an inverted U-shaped relationship between GDP and urban CO_2_ emissions from the transportation, residential, and industrial sectors. Nugraha et al. [33] discovered that the rise in the added value of the agriculture sector would reduce CO_2_ emissions. Anwar et al. [34] demonstrated that the relationships between the added value of primary industry and CO_2_ emissions were different among countries with different income levels. Using an autoregressive distributed lag model, Anwar et al. [35] proved that industrial value added per capita had a positive relationship with CO_2_ emissions. However, Lin et al. [36] showed that the added value of industry had an inverse effect on CO_2_ emissions in Nigeria.

Although there are a large number of studies on the effects of urban governance elements on CO_2_ emissions, most research paid attention to the correlations between governance factors and CO_2_ emissions. However, the research on the influence mechanism between urban governance factors and CO_2_ emissions is not deep enough. Most of the non-linear relationships between the governance elements (except for GDP and per capita GDP) and CO_2_ emissions have not yet been explored. The main influencing factors of CO_2_ emissions in specific sectors have not been clarified. Moreover, more attention has been paid to the independent effects of individual governance element on CO_2_ emissions. However, the overall effects of governance factors on CO_2_ emissions have not yet been examined because of the lack of comprehensively considering urban governance elements. So it is hard to translate the impact evaluation of governance factors on CO_2_ emissions into practical applications.

### 2.2 Carbon dioxide emissions prediction

Various modeling methodologies have been adopted to forecast CO_2_ emissions. The most convenient methods are time series forecasting models, such as autoregressive integrated moving averages (ARMIA) and gray models. For example, Hamzacebi et al. [37] predicted the energy-related CO_2_ emissions of Turkey using a gray prediction model. Malik et al. [38] used a ARMIA model to forecast CO_2_ emissions for Pakistan. These methods only consider the roles of time factors in predicting CO_2_ emissions but not the specific factors affecting CO_2_ emissions, so the methods cannot describe the influence mechanism of CO_2_ emissions.

In order to take governance elements into account, some scholars have used regression models, such as multiple linear regression models, logistic models, and vector auto-regressive models, which can capture the relationships between CO_2_ emissions and governance variables. For instance, Jiang et al. [39] considered the effect of temperature, and CO_2_ efflux was estimated using different regression methods in static chamber observation from an alpine meadow on the Qinghai-Tibetan Plateau. Du et al. [40] consider about the variables of GDP, GDP energy intensity and energy carbon intensity, and forecast CO_2_ emissions of provinces in 2050 China based on logistic model. Cui HR et al. [41] analyzed the dynamic relationship between energy, economy and the environment, and predicted energy-related CO_2_ emissions from 2016 to 2023. However, These studies only consider socio-economic factors. Moreover, the regression models with simple structures have limitations such as easy under fitting, sensitivity to outliers, and low accuracy in non-linear data processing [42].

There are many non-linear relationships between governance factors and CO_2_ emissions. In order to reveal the non-linear relationship and improve the accuracy of prediction, many academics have begun to employ artificial intelligence models such as artificial neural networks [43], wavelet neural network predictive models [44], genetic algorithm support vector machines [45], and least squares support vector machines [46]. However, these studies mainly consider about the socio-economic elements. Other urban governance elements such as urban space and facilities which have been confirmed to have significant effects on CO_2_ emissions were not included. Meanwhile, in these prediction studies, the relationship between governance elements and CO_2_ emissions have not been revealed, the application in urban governance has certain limitations.

## 3 Methodology

### 3.1 Data

#### 3.1.1 Data sources

Cities are the main sources of CO_2_ emissions in China [47]. The data in this study are derived from the county-level cities which are the administrative units of the country. In Chinese administrative management, the county-level cities reflect the small cities. Their large populations, wide land coverage, and high proportions of total CO_2_ emissions give them key roles to play in CO_2_ emission mitigation, while the existing research is still insufficient. To ensure the universality and accuracy of the prediction model, it is necessary to use the data of several years to train the model. Since only 2010, 2012 and 2015 city CO_2_ emissions data are available, we finally selected the statistics from these years of 1903 county-level cities as the experimental data. The governance variable data of each department were taken from the China County Statistical Yearbooks of the same years. The CO_2_ emission data of the residential, industrial, and transportation sectors were obtained from the China City Carbon Dioxide Emissions Datasets of the same years.

#### 3.1.2 Data processing

Because of the lack of authoritative data on residential, industrial, and transportation emissions at the county level, this study used a top-down distribution method to estimate the CO_2_ emissions of county-level cities [48]. The steps taken are as follows.

First, the total CO_2_ emissions (CE) are composed of three major parts: residential CO_2_ emissions (RCE), industrial CO_2_ emissions (ICE), and transportation CO_2_ emissions (TCE):

(1) $${CE}_{m}={RCE}_{m}+{ICE}_{m}+{TCE}_{m}$$

Second, the CO_2_ emissions of prefecture-level cities are decomposed to estimate the CO_2_ emissions of each sector in the counties. Beginning with the first term, which can be expressed as:

(2) $${RCE}_{m}={RCE}_{n}\times a_{m},a_{m}=\frac{P_{m}}{P_{n}}$$

where RCE_m_ is the total RCE of county m, RCE_n_ is that of city n where m county is located, a_m_ is the allocation coefficient, P_m_ is the total population of county m, and P_n_ is the total population of city n.

(3) $${ICE}_{m}={ICE}_{n}\times b_{m},b_{m}=\frac{V_{m}}{V_{n}}$$

where ICE_m_ is the total ICE of county m, ICE_n_ is that of city n where m county is located, b_m_ is the distribution coefficient, V_m_ is the total industrial output value of county m, and V_n_ is the total industrial output value of city n.

(4) $${TCE}_{m}={TCE}_{n}\times c_{m},c_{m}=\frac{R_{m}}{R_{n}}$$

where TCE_m_ is the total TCE of county m, TCE_n_ is that of city n where m county is located, c_m_ is the distribution coefficient, R_m_ is the total road length in county m, and R_n_ is the total road length in city n.

To verify the credibility of the data, the correlation analysis and simple linear regression analysis methods are used to compare the ${CE}_{m}$ obtained from the above steps with the grid total CO_2_ data from the High Spatial Resolution Greenhouse Gas Online Platform (https://wxccg.cityghg.com/geo) and the County-level CO_2_ Emissions Data in China released by J Chen et al. [49] in Scientific Data. The results showed that the correlation coefficient were all greater than 0.7, and the significance of regression analysis and correlation analysis were all 0.000, which means the research data is reasonable.

#### 3.1.3 Variables

To predict CO_2_ emissions by using urban governance elements, it is necessary to select appropriate governance variables. We referred to the existing literatures and the important variables involved in the county-level cities planning policies, selected the variables with significant correlation with CO_2_ emissions (p < 0.05), and removed the variables with duplication. Finally, 14 variables shown in Table 2 were selected. They cover almost all aspects of Economic & Social Development Planning and Land & Space Planning, including city size & structure, economic development & industrial structure, municipal utility facilities, road traffic facilities and public service facilities. Among them, we extracted 10 variables directly from relevant literature: built-up area, land urbanization rate, population, GDP, added value of primary industry, added value of secondary industry, residential density, coverage rate of population with access to gas, coverage of central heating, and density of road networks. Meanwhile, as heating facilities consume large amounts of energy and thus produce RCE [15], the density of heating pipelines was included in the governance factors. Considering the effects of allocation strategies for public service facilities [50] and infrastructure construction levels [51] on TCE into account, we included average service area of the park, average number of beds served, and pavement area ratio.

**Table 2:** Definitions of all variables in the study.

Category	Variable	Measurement unit	Sector	Reference
City size & structure	Built-up areas	km^2^	RCE, ICE, TCE	[18,25,28]
	Land urbanization rate	%	RCE, ICE, TCE	[17,24,27]
	Population	10,000 people	RCE, ICE, TCE	[17,25,26]
	Residential density	10,000 people/km^2^	RCE	[19,18]
Economic development & industrial structure	GDP	hundred million RMB	RCE, ICE, TCE	[31,32]
	Added value of primary industry	million RMB	RCE, ICE, TCE	[33,34]
	Added value of secondary industry	million RMB	RCE, ICE, TCE	[21,35,36]
Municipal utility facilities	Coverage rate of population with access to gas	%	RCE	[41]
	Coverage of central heating	%	RCE	[41]
	Density of heating pipelines	km/km^2^	RCE	[15]
Road traffic facilities	Density of road network	km/km^2^	TCE	[28]
	Pavement area ratio	%	TCE	[51]
Public service facilities	Average service area of the park	km^2^	TCE	[29]
	Average number of beds served	person	TCE	[29]

### 3.2 Method

Random forest model is a combined classification method that outperforms single algorithms in terms of accuracy. It have strong fitting ability and complex model structure to capture the non-linear and non-parametric relationship between CO_2_ emissions and governance elements. And the model training speed is fast and efficient in processing large data sets. At the same time, the model has strong interpretability. Different from other “black-box" artificial intelligence models, random forest can evaluate the contributions of predictor variables on CO_2_ emissions, and reveal the impact mechanism. At present, random forest model has been successfully used to the prediction of gaseous pollutants, such like NO_2_ [52]. This study intends to propose a random forest model based on integrated urban governance elements to predict CO_2_ emissions and further analyze the relationship between governance elements and CO_2_ emissions.

**Fig. 1:**
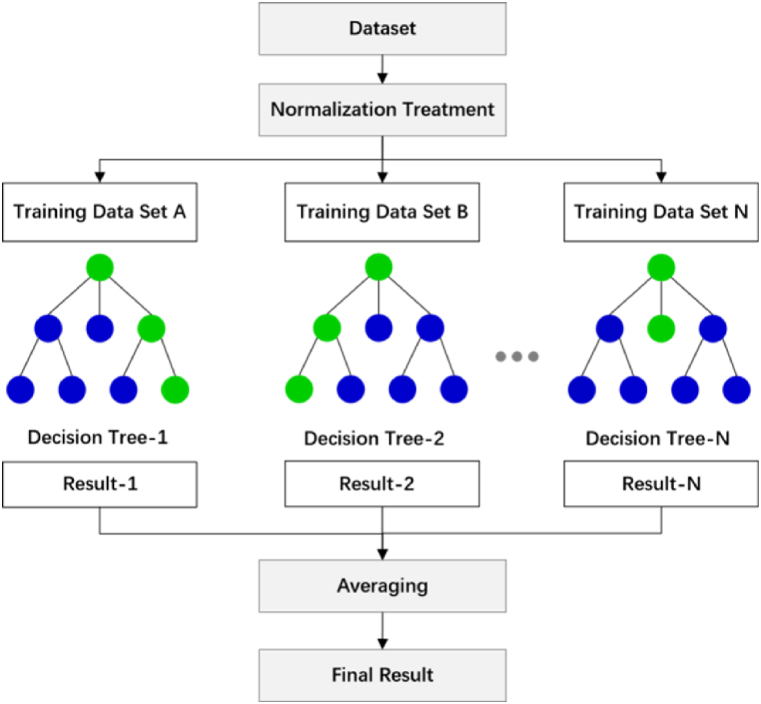
Schematic diagram of random forest.

Proposed by Leo Breiman [53], random forest is an integrated learning method that takes a decision tree as the basic unit and combines bagging with classified regression trees. During the training process, a bootstrap re-sampling technique is used to randomly select k samples from the original training set for constructing k decision trees with weak performance [53]. Every decision tree can grow without constraint and pruning while staying independent without correlation. Each decision tree generates the input values of the model and average or majority voting determines the output values [54]. Random forest performs well in processing the highly nonlinear relationship between a set of inputs and outputs, which is suitable for establishing regression models [17]. It can also evaluate the importance of features [55]. In our study, random forest was used to explore not only predictions of CO_2_ emissions, but also the importance of urban governance factors. The action flow is shown in Fig. 1, and the basic formulas are as follows.

(1) For any sample X, having P sub-models and will generate P prediction values. Suppose that the predicted value of the kth sub-model is ${\hat{Y}}_{k}$, the total model ${\hat{Y}}_{E}$ will produce results by simple averaging [56]:

$${\hat{Y}}_{E}=\frac{1}{P}\sum_{k=1}^{P}{\hat{Y}}_{k}$$

(2) The feature importance score of random forest mainly evaluates the degree of the contribution of each feature that participates in the operation of the decision tree. The importance analysis of characteristics is mainly based on out-of-bag data (OOB), which are a data set composed of the sample points that are not selected each time the model performs a random sampling with replacement on the training set. The importance of the variables is measured by the percentage increase in mean squared error（IncMSE%） of the OOB data. IncMSE% means a decrease in the accuracy of the target prediction after the variables are removed, so more important variables have higher IncMSE%. For a decision tree, the corresponding variables of the OOB data were put into the decision tree before and after scrambling, then their IncMSE% was calculated. Suppose there are N trees in the forest, then the IncMSE% for the K tree is:

$$IncMSE\%\left(i\right)=\sum_{K=1}^{N}\frac{\left({OOB}_{k2}-{OOB}_{k1}\right)}{{OOB}_{k1}}\times 100\%\text{,}$$

where i is a variable under consideration, ${OOB}_{k1}$ is the OOB error before disruption, and ${OOB}_{k2}$ is the OOB error after disruption. For n trees, if i has no influence on the result of the decision tree after the scrambling of the OOB data and the difference of the mean square error after scrambling is very small, then i is not important [57].

## 4 Results and discussions

### 4.1 Contributions of independent variables

Table 3 describes the combined contribution of the independent variables to RCE, ICE and TCE in 2010, 2012 and 2015. According to Table 3, the governance variables contributing greatly to RCE are density of heating pipelines (44.32%) and population (39.82%). Other elements with more contributions are coverage rate of population with access to gas (31.17%), coverage of central heating (29.81%) and built-up areas (29.74%). The importance of the remaining urban governance elements is living density (23.73%), added value of secondary industry (21.69%), land urbanization rate (21.63%), GDP (17.41%), and added value of primary industry (15.72%). The strong effects of heating pipeline density on CO_2_ emissions show that heating energy consumption is an important part of residential energy consumption and heating CO_2_ emissions are also an important source of RCE. The strong effects of population on RCE indicate that population growth will increase the consumption of energy used for daily life and produce high CO_2_ emissions.

**Table 3:** Relative contributions of independent variables to CO_2_ emissions.

Independent variables	RCE		ICE		TCE	
	IncMSE%	Rank	IncMSE%	Rank	IncMSE%	Rank
Built-up areas	29.74	5	18.75	5	25.37	4
Land urbanization rate	21.63	8	18.62	6	37.69	1
Population	39.82	2	18.80	4	23.06	6
GDP	17.41	9	24.73	2	31.13	2
Added value of primary industry	15.72	10	23.70	3	23.74	5
Added value of secondary industry	21.69	7	34.38	1	22.31	7
Density of road network	–	–	–	–	26.58	3
Living density	23.73	6	–	–	–	–
Coverage rate of population with access to gas	31.17	3	–	–	–	–
Coverage of central heating	29.81	4	–	–	–	–
Density of heating pipelines	44.32	1	–	–	–	–
Pavement area ratio	–	–	–	–	11.06	10
Average service area of the park	–	–	–	–	21.41	8
Average number of beds served	–	–	–	–	12.63	9

The most important variables for ICE are the added value of secondary industry (30.04%), GDP (24.73%), the added value of primary industry (23.70%), and population (18.80%). The strong effects of the added value of secondary and primary industry and GDP indicate that the development of industry could result in high energy consumption and CO_2_ emissions in the industrial sector. Among them, the second industry has the most influence. It also indicate that adjusting the industrial structure could be an effective method to control ICE. In addition, the significant effect of population on ICE shows that population growth would increase the energy demand of the industrial sector.

Land urbanization rate (37.69%), GDP (31.13%), density of road networks (26.58%) and built-up areas (25.37%) are the most important governance factors of TCE. The importance of the remaining elements of urban governance are added value of primary industry (23.74%), population (23.06%), the added value of secondary industry (22.31%), average service area of the park (21.41%), average number of beds served (12.63%) and pavement area ratio (11.06). The strong effects of land urbanization rate and built-up areas on TCE imply that the expansion of urban land would affect residents' travel needs and travel convenience. The strong effects of GDP on TCE mean that the development of urban economies would increase energy consumption by the transportation sector. The important contributions of the density of road networks to TCE indicate that the construction level of transportation facilities is closely related to TCE.

### 4.2 Non-linear effects of key independent variables

**Fig. 2:**
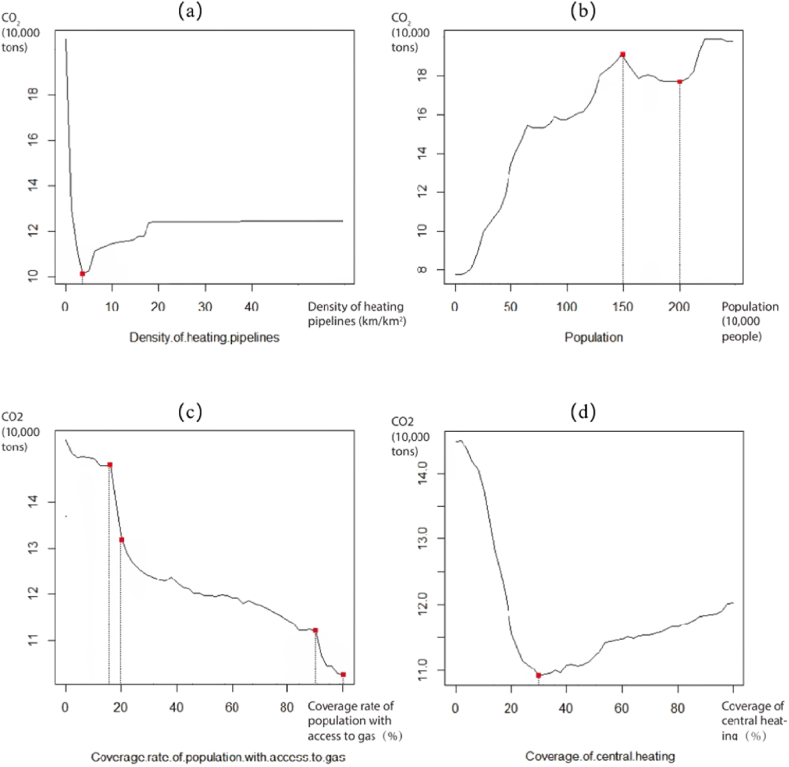
Non-linear relationships between governance elements and RCE. Note: (a) Non-linear relationship between density of heating pipelines and RCE, (b) Non-linear relationship between population and RCE, (c) Non-linear relationship between coverage rate of population with access to gas and RCE, (b) Non-linear relationship between coverage of central heating and RCE.

We select the four variables with the highest contributions in each CO_2_ emission sector for non-linear mechanism analysis. Fig. 2 illustrates the effects of key urban governance variables on RCE. With improvements in heating pipeline density, RCE first decreases. When heating pipeline density is within 3–19 km/km^2^, RCE shows a sustained increase. Hence, if the heating pipe density is too low, then residents’ demand for heating facilities, such as air-conditioning, that consume much energy would be high and result in high CO_2_ emissions. So, heating pipeline density should be controlled at about 3 km/km^2^. Population has a increasing effect on RCE, which is consistent with the conclusions of other studies. But it is worth noting that this change fluctuates when the population is between 1.5 and 2 million in this research. This may be explained that the dense population tends to choose smaller houses, thus reducing energy consumption. However, when the population is too dense, the heat island effect will make residents increase energy consumption. The coverage rate of the population with access to gas has a restraining effect on RCE. The suppressive effects of the gas penetration rate, especially when the rate are 15%–20% and 90%–100%, indicating that the popularization of clean energy could reduce the use of fossil fuels. The effects of the coverage of central heating on RCE are inhibitory within the range of 0%–29%. However, domestic CO_2_ emissions start to rise slowly as the coverage of central heating reaches 29%. Although the coverage of central heating is more favorable to some extent than distributed heating methods for low CO_2_ emissions, the demand for energy is gradually increasing with the development of central heating.

**Fig. 3:**
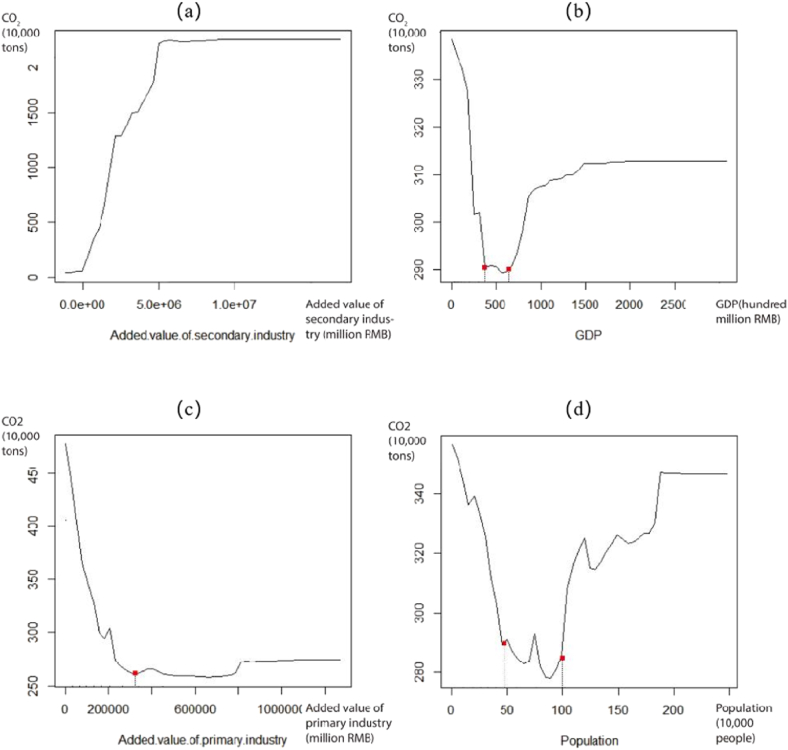
Non-linear relationships between governance elements and ICE. Note: (a) Non-linear relationship between added value of secondary industry and ICE, (b) Non-linear relationship between GDP and ICE, (c) Non-linear relationship between added value of primary industry and ICE, (b) Non-linear relationship between population and ICE.

As shown in Fig. 3, the added value of secondary industry has a positive effect on ICE, which indicates that the development of secondary industry would produce more CO_2_ emissions. On the contrary, with the increase of the added value of primary production, ICE gradually decreases, which is consistent with the existing research results. It can be explained that compared with the secondary industries with high energy consumption, the primary industry consumes less energy. GDP signifies a “U-type” nexus on CO_2_ emissions, and the level of ICE is at its lowest when GDP is at 400–600 hundred millions RMB, which is inconsistencies with studies of large cities [32]. This change probably can be explained in relation to the curve of the added value of primary and secondary industry. For Chinese county-level cities, their economic development is relatively slow at the beginning and they rely mainly on the primary industry. With the improvement of the agriculture technology, the added value of the primary industry increases and CO_2_ emissions decreases. However, with the further rapid development of the economy, the rapid expansion of the secondary industry has caused a large amount of CO_2_ emissions. But this trend will slow down gradually with the improvement of industrial technology and the development of tertiary industry. As for the effects of population, ICE drops sharply at first, then reaches the lowest level within the range of 500,000 to 1 million people, beyond which ICE shows an upward trend. When the population size approaches 1.8 million people, ICE rises sharply, followed by a continuous increase before reaching 2 million people. This may be related to the relationship between population and industry type. Green industries need a certain labor base, but labor-intensive industries are often energy-intensive industries, which can generate a large amount of CO_2_ emissions. With the increase in the proportion of the added values of the primary and secondary industries, ICE first decreases sharply, then increases gently. If the added value ratio of the primary and secondary industries is controlled at 0.5, then the level of ICE is at its lowest.

**Fig. 4:**
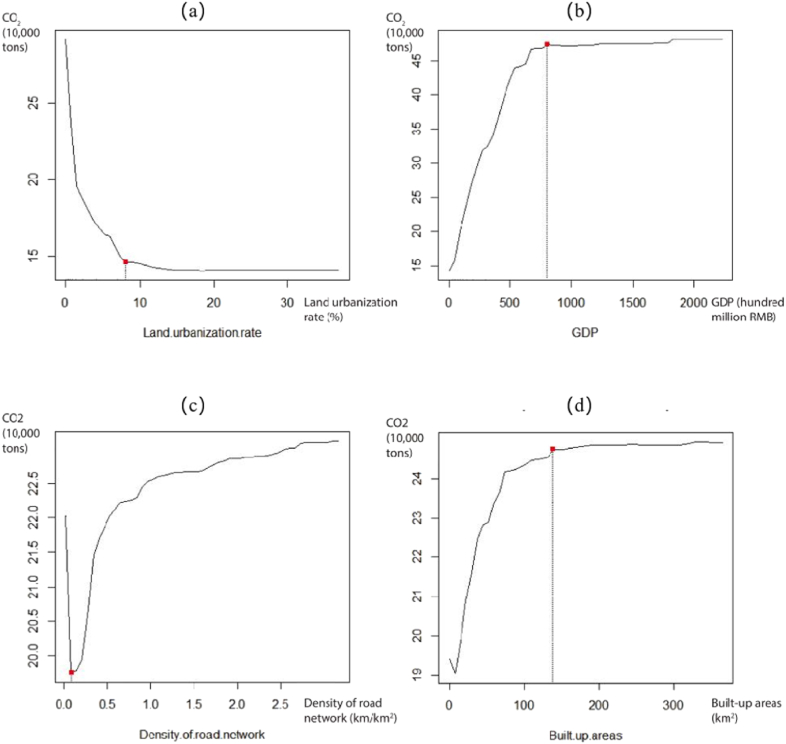
Non-linear relationships between governance elements and TCE. Note: (a) Non-linear relationship between land urbanization rate and TCE, (b) Non-linear relationship between GDP and TCE, (c) Non-linear relationship between density of road network and TCE, (b) Non-linear relationship between built-up areas and TCE.

In Fig. 4, land urbanization rate has an inhibitory effect on TCE, which may be due to the convenience of the road increases the car travel. This change is most obvious before the land urbanization rate to 8%. We investigated the effects of GDP and built-up areas on TCE, which generally experiences a process from rapid to slow. When the built-up areas reach 150 km^2^ and GDP reaches RMB700 hundred million, the influence degree of these governance elements remains stable at its maximum, thus indicating that urban sprawl and economic growth would increase residents’ daily travel distances and probabilities of car usage. The effects of road network density on TCE show a trend of first decreasing, then increasing, which reflects the combined effect of city size and road length on CO_2_ emissions. At the beginning, the layout of the road network has greatly improved the accessibility of traffic, thereby reducing CO_2_ emissions. However, when the construction of urban road network exceeds the demand for basic accessibility, it will greatly enhance the willingness of residents to travel by car, and even cause traffic congestion, thus increasing CO_2_ emissions.

### 4.3 Simulation and results

To test the discriminant ability of random forest for new samples, the data for 2010, 2012, and 2015 of 1903 county-level cities were divided into two data sets: training and test samples. Data samples from 1500 counties were randomly selected as the training samples and the remaining 402 samples were used as the test samples. In the test data set, the average correlation coefficients of RCE, ICE, and TCE are 0.9327, 0.9674, and 0.9431, respectively. The results show that the random forest algorithm has good discriminant ability for new samples.

To further test the rationality of random forest in predicting CO_2_ emissions from governance factors, we contrasted the results of random forest with those of a linear regression model, lasso regression model, and support vector regression machine. The data in 2018 of the governance factors and CO_2_ emissions for 1902 counties were used as test sets to examine the predictive abilities of the four models. The data on the governance factors of each department were fed into each model to calculate the predicted CO_2_ emissions of each department in all counties. The deviations between the predicted values and the actual values reflected the performance of the models. The root mean square error (RMSE) and root average squared error (RMAE) were the variables chosen to represent the deviations. Low values for both variables refer to smaller deviations, which indicate the better fitting of a model.

**Fig. 5:**
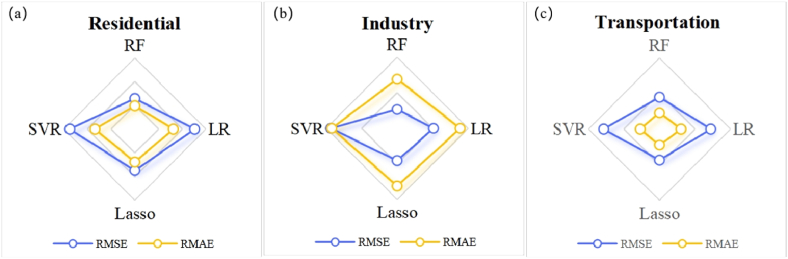
Comparison of predicted values. Note: (a) Comparison of predicted values of residential sector, (b) Comparison of predicted values of industry sector, (c) Comparison of predicted values of transportation sector.

According to Table 4 and Fig. 5, RMSE and RMAE of the random forest algorithm are significantly smaller than those of the other models, which indicates that the predictive ability of the random forest model is better and proves a complex influence mechanism between the governance factors and CO_2_ emissions. Such a mechanism would be difficult for traditional linear models to quantify the relationship between the factors and emissions.

**Table 4:** RMSE and RMAE of the four models.

Governing Sector	Random Forest (RF)		Linear Regression (LR)		Lasso Regression		Support Vactor Regression (SVR)	
	RMSE	RMAE	RMSE	RMAE	RMSE	RMAE	RMSE	RMAE
Residential	64	4.9	126	8.1	87	6.9	137	8.4
Industry	52,741	138	102,374	177	91,012	162	187,312	184
Transportation	178	9.1	288	12.1	173	8.9	314	10.8

Comparing with the prediction models in existing studies, our model based on the complex and interpretable effects of urban governance elements on CO_2_ emissions. It runs effectively on big data sets and is applicable to big data sets in county-level cities in China. Furthermore, it have better prediction performance of lower RMSE and RMAE, and can further reveals the complex non-linar quantitative relationship between urban governance elements and sectoral CO_2_ emissions, which is conducive to more specific governance. However, energy consumption and the relationship between governance factors and CO_2_ emissions may change over time, but this model cannot consider the impact of time dimension changes.

### 4.4 Applications of predictive model

To translate the theoretical results into practical applications, this study established an intelligent platform based on the above methods for predicting CO_2_ emissions from urban governance elements. Urban CO_2_ emission data can be directly generated by inputting the planning data of recent or future governance variables of a city into this platform. Taking the Zhengding county-level city as an example, the platform predicts the CO_2_ emission levels of the region by using the different planning schemes of governance variables for 2030 and selecting the optimal planning scheme. The corresponding scenario models of Plan A, B and C are factor regulation scenario, baseline development scenario and radical development scenario respectively. The baseline development scenario represents the continuation of the past development characteristics. The element regulation scenario represents the optimization and adjustment of various urban governance elements. And the radical development scenario represents the weakening of the management of governance elements. The value of each governance element under baseline development scenario model are set according to the overall situation and annual average rate of change of the element in recent ten years, and other scenario settings are adjusted within a certain range according to relevant policies and plans of the Zhengding County. As shown in Table 5, Plan A (factor regulation scenario) produces the lowest levels of CO_2_ emissions and hence is an effective scheme for mitigating the emissions.

**Table 5:** Planning schemes for governance elements.

Variable	Plan A	Plan B	Plan C
Built-up areas	140	145	150
Land urbanization rate	26	28	30
Population	52	56	58
GDP	300	330	350
Added value of primary industry	350,000	380,000	400,000
Added value of secondary industry	1,200,000	1,500,000	1,800,000
Density of heating pipelines	1.2	1.5	1.8
Density of road network	1.8	2	2.2
Pavement area ratio	30	25	20
Living density	4	4.2	2.2
Average service area of the park	12	8	6
Average number of beds served	0.07	0.05	0.03
Coverage of central heating	100	100	100
Coverage rate of population with access to gas	100	100	100
Total CO_2_ emissions	709.97	1015.09	1197.83

## 5 Conclusions and policy implications

Using random forest, this study examined the quantitative relationship between urban governance elements and CO_2_ emissions, and the prediction platform has established. The conclusions of this paper are as follows.

(1) The most important governance factors of RCE are density of heating pipelines and population, following are coverage rate of population with access to gas and coverage of central heating, indicating the construction and control of municipal public facilities are crucial. For ICE, the added value of secondary industry, GDP and the added value of primary industry play important roles, indicating the economic development & industrial adjustment have big effect. For TCE, the key governance factors are land urbanization rate, GDP, density of road networks and built-up areas. Which means the city size & structure element and the allocation of road transport facilities have an important impact on CO_2_ emissions.
(2) There are many non-linear relationship between governance elements and CO_2_ emissions of various sectors. For RCE, both density of heating pipelines and coverage of central heating indicate a “U-type” nexus. Population has a increasing effect on RCE, while the coverage rate of population with access to gas has a opposite effect. For the industrial sector, the added value of the secondary industry has a significant positive impact on ICE, while the impact of the primary industry is the opposite. Population and GDP display a “U-type” connection with ICE. As for TCE, the land urbanization rate has negative effect while the built-up areas and GDP have positive effects. The density of road networks shows a “U-type” nexus with TCE.
(3) Random forests can be used to predict CO_2_ emissions and perform well. Using the intelligent platform established in this paper for predicting CO_2_ emissions from urban governance factors, decision-makers can obtain CO_2_ emission data only by inputting the data of various governance variables according to policy planning. This platform intuitively reflects how CO_2_ emission levels increase or decrease according to changes in the governance factor variables.

The research findings have the following policy and operational implications for the government. For residential sector, governments should pay much attention to municipal public facilities governance. Considering the U-shaped influence mechanism of density of heating pipelines and coverage of central heating, the authorities should rationally control the construction density of heating infrastructure and coverage of central heating according to residents' demand for heating. So that avoiding the oversupply of facilities and energy while improving the utilization rate of heat energy. Meanwhile, considering the inhibition effect and importance of coverage rate of population with access to gas on CO_2_ emissions, the authorities should vigorously develop clean energy and encourage residents to switch from heating with fossil fuels to the use of clean energy. Especially when the coverage rate of population with access to gas in county-level cities is close to 15% or 90%, the government should make more efforts taking measure. As in this range, a small increase can have a big return.

As for industrial sector, economic development & industrial adjustment need urgent attention by decision-makers. For county-level cities, due to the relatively backward level of industrialization and the low level of industrial technology, the growth of secondary industry has a significantly positive effect on CO_2_ emissions. Therefore, the government should focus on industrial restructuring, vigorously develop green environmental protection industries, and try to achieve decoupling between economic development and CO_2_ emissions. The authorities should not blindly learn from big cities to pursue secondary industrial development, but should encourage qualified areas to develop tourism, cultural industry, ecological agriculture etc. In addition, for county-level cities with good geographical advantages and certain industrial base. The government are supposed to eliminate traditional backward industries, catalyze the modernization and transformation of traditional manufacturing industry, and develop high-end intelligent industries.

When it comes to transportation sector, the government should mainly focus on the control of urban expansion, and the allocation of road traffic facilities. The governments should control the sizes of the built-up areas, adopt a compact urban pattern, thus reduce potential private car transportation requirements, and reduce CO_2_ emissions from long-distance travel caused by the unlimited and scattered expansion of land. For road traffic facilities, considering the U-shaped influence mechanism of road network density, decision-makers should make overall plans for green transportation and construction based on comprehensive travel demand analysis and transportation planning. While constructing road traffic facilities, it must be noticed to increase the degree of coupling between urban population density, urban form, and traffic organization, and systematically plan the densities of road networks, thus avoid increases in CO_2_ emissions caused by unreasonable transportation system planning. Furthermore, urban public service facilities should improve equality and convenience to reduce the TCE caused by long-distance travel.

However, this study also have certain limitations which need to be improved in future research. First, the study is limited by incomplete statistical data because some urban governance factors may have been overlooked and therefore not included. Future research can focus on establishing more comprehensive relationships between governance factors and CO_2_ emissions. In addition, we did not consider the impact of the time dimension and ignored the change of the relationship between governance factors and CO_2_ emissions. Moreover, our study did not consider about the regional heterogeneity. In fact, China is so vast, and the natural conditions, resource endowments and cultural beliefs of different regions vary greatly, which may effect the results of the study. Future studies can further carry out specific analysis of different regions.

## Author contribution statement

He Zhang: conceived and designed the experiments; Jingyi Peng: conceived and designed the experiments, performed the experiments, analyzed and interpreted the data, contributed reagents, materials, analysis tools or data and wrote the paper.

Rui Wang: conceived and designed the experiments.

Mengxiao Zhang: conceived and designed the experiments, performed the experiments, analyzed and interpreted the data and wrote the paper.

Chang Gao: contributed reagents, materials, analysis tools or data.

Yang Yu: contributed reagents, materials, analysis tools or data.

## Data availability statement

The authors do not have permission to share data.

## Declaration of competing interest

The authors declare that they have no known competing financial interests or personal relationships that could have appeared to influence the work reported in this paper.
